# Preoperative warming combined with intraoperative skin-surface warming does not avoid hypothermia caused by spinal anesthesia in patients with midazolam premedication

**DOI:** 10.1590/S1516-31802007000300004

**Published:** 2007-05-03

**Authors:** Simone Maria D’Angelo Vanni, Yara Marcondes Machado Castiglia, Eliana Marisa Ganem, Geraldo Rolim Rodrigues, Rosa Beatriz Amorim, Fábio Ferrari, Leandro Gobbo Braz

**Keywords:** Spinal anesthesia, Hypothermia, Thermoregulation, Equipment and supplies, Midazolam, Raquianestesia, Hipotermia, Termorregulação, Equipamentos e provisões, Midazolam

## Abstract

**CONTEXT AND OBJECTIVE:**

Inadvertent perioperative hypothermia is common during spinal anesthesia and after midazolam administration. The aim of this study was to evaluate the effects of intraoperative skin-surface warming with and without 45 minutes of preoperative warming in preventing intraoperative and postoperative hypothermia caused by spinal anesthesia in patients with midazolam premedication.

**DESIGN AND SETTING:**

Prospective and randomized study at Hospital das Clínicas, Universidade Estadual Paulista, Botucatu.

**METHODS:**

Thirty patients presenting American Society of Anesthesiologists (ASA) physical status I and II who were scheduled for elective lower abdominal surgery were utilized. The patients received midazolam premedication (7.5 mg by intramuscular injection) and standard spinal anesthesia. Ten patients (Gcontrol) received preoperative and intraoperative passive thermal insulation. Ten patients (Gpre+intra) underwent preoperative and intraoperative active warming. Ten patients (Gintra) were only warmed intraoperatively.

**RESULTS:**

After 45 min of preoperative warming, the patients in Gpre+intra had significantly higher core temperatures than did the patients in the unwarmed groups (Gcontrol and Gintra) before the anesthesia (p < 0.05) but not at the beginning of surgery (p > 0.05). The patients who were warmed intraoperatively had significantly higher core temperatures than did the patients in Gcontrol at the end of surgery (p < 0.05). All the patients were hypothermic at admission to the recovery room (T_CORE_ < 36° C).

**CONCLUSIONS:**

Forty-five minutes of preoperative warming combined with intraoperative skin-surface warming does not avoid but minimizes hypothermia caused by spinal anesthesia in patients with midazolam premedication.

## RESUMO

**CONTEXTO E OBJETIVO:**

Hipotermia inadvertida no perioperatório é freqüente durante anestesia subaracnóidea e após a administração de midazolam. O objetivo foi avaliar os efeitos do aquecimento da pele no intra-operatório, associado ou não ao aquecimento da pele durante o período de 45 minutos no pré-operatório, na prevenção de hipotermia intra- e pós-operatória determinada pela anestesia subaracnóidea em pacientes com medicação pré-anestésica com midazolam.

**TIPO DE ETUDO E LOCAL:**

Estudo prospectivo e aleatório, realizado no Hospital das Clínicas, Universidade Estadual Paulista (Unesp), Botucatu, SP.

**MÉTODOS:**

O estudo foi realizado em 30 pacientes com estado físico ASA (da Sociedade Norte-americana de Anestesiologistas) I e II submetidos à cirurgia eletiva do abdômen. Como medicação pré-anestésica, utilizou-se o midazolam, 7,5 mg via intramuscular (IM) e anestesia subaracnóidea padrão. Em 10 pacientes (Gcontrole) utilizou-se isolamento térmico passivo; 10 pacientes (Gpré+intra) foram submetidos a aquecimento ativo no pré- e intra-operatório; e 10 pacientes (Gintra) foram aquecidos ativamente somente no intra-operatório.

**RESULTADOS:**

Após 45 minutos de aquecimento no pré-operatório, os pacientes do Gpré+intra apresentaram temperatura central mais elevada em relação aos dos grupos não aquecidos antes da anestesia (p < 0,05) mas não no início da cirurgia (p > 0,05). Os pacientes que receberam aquecimento no intra-operatório apresentaram temperatura central mais elevada no final da cirurgia em relação aos de Gcontrole (p < 0,05). Todos os pacientes estavam hipotérmicos na admissão da sala de recuperação pós-anestésica (temperatura central < 36° C).

**CONCLUSÕES:**

45 minutos de aquecimento no pré-operatório combinado com aquecimento no intra-operatório não evita, mas minimiza a ocorrência de hipotermia determinada pela anestesia subaracnóidea em pacientes que receberam midazolam como medicação pré-anestésica.

## INTRODUCTION

Inadvertent perioperative hypothermia is common during neuraxial (spinal and epidural) anesthesia,^1–3^ which may be as severe as during general anesthesia.^1–3^ Three major factors contribute towards core hypothermia during neuraxial anesthesia: 1) internal redistribution of heat from the core of the body to peripheral tissues; 2) heat loss to the environment; and 3) inhibition of vasomotor and shivering responses.^4,5^ Body temperature is often not monitored during regional anesthesia.^6^ One insidious aspect of hypothermia in neuraxial anesthesia is that not only does inhibition of autonomic thermoregulatory control take place,^5^ but also there is inhibition of behavioral control.^7^ As a result, surgical patients who are given epidural or spinal anesthesia frequently have hypothermia that is rarely detected by either the patient or the anesthesiologist.^7,8^

Intraoperative hypothermia often induces adverse outcomes, including coagulopathy and increased blood loss,^9^ surgical wound infections and prolonged hospitalization,^10^ intraoperative and postoperative cardiac events,^11^ discomfort^12^ and postoperative shivering.^13^

One to two hours of active skin-surface warming before inducing general^14,15^ and epidural anesthesia^16^ minimizes intraoperative hypothermia. Such prolonged prewarming is, however, impractical in most hospitals. It has been demonstrated that 30 minutes to one hour of forced-air warming increases peripheral tissue heat content by more than the amount normally redistributed during the first hour of anesthesia.^17^

Midazolam is a commonly used sedative agent that is often used for premedication. One study^18^ has shown that midazolam impedes thermoregulatory vasoconstriction and produces core-to-peripheral heat distribution in a dose-dependent manner. Therefore, its use for premedication may affect temperature control during general anesthesia.^19^ However, the effectiveness of preoperative warming combined with intraoperative warming for maintaining core temperature during general anesthesia among patients with midazolam premedication was recently demonstrated.^15^ The hypothesis of our study was that preoperative warming combined with intraoperative warming would maintain the core temperature during spinal anesthesia, among patients with midazolam premedication.

## OBJECTIVE

The aim of this study was to evaluate the effect of intraoperative warming with and without 45 minutes of preoperative skin-surface warming using a forced-air warming system, during lower abdominal surgery under spinal anesthesia, with regard to preventing intraoperative and postoperative hypothermia in patients who received preoperative administration of midazolam.

## METHODS

The study protocol was approved by the Hospital Ethics Committee, and written informed consent was obtained from 30 patients presenting American Society of Anesthesiologists (ASA) physical status I and II who were scheduled for elective lower abdominal surgery lasting at least one hour at Hospital das Clínicas, Universidade Estadual Paulista (Unesp), Botucatu, São Paulo, Brazil, which is a public tertiary teaching hospital. None of the patients were obese, febrile or taking vasoactive drugs, or had any history of endocrine disease.

The patients received midazolam (7.5 mg by intramuscular injection) 30 minutes before their admission to the operating room. All patients had fasted for at least eight hours. They all received an intravenous (IV) infusion of lactated Ringer's solution (8 to 10 ml.kg^-1^.h^-1^) into a right antecubital vein, as IV maintenance fluid. Fluids were kept at operating room temperature before infusion. The patients were monitored by electrocardiography (EKG) and pulse oximetry, and using noninvasive blood pressure and temperature measurements.

All temperatures were measured using thermocouple probes (Mon-a-Therm, Mallinckrodt Medical, United States). Ambient temperature (T) was measured by a thermocouple probe placed near the patient, but away from any heat-generating equipment. Core temperature (T_CORE_) was measured using a cotton-tipped probe that was positioned adjacent to the tympanic membrane. The external auditory channel was packed with cotton for insulation. Skin temperatures were measured at four skin-surface sites: chest (T_CHEST_), left upper arm (T_ARM_), left lateral mid-thigh (T_THIGH_), and left mid-calf (T_CALF_). Mean skin temperatures (MST) were calculated according to the formula given by Ramanathan:^20^


$$\text{MST}=0.3\;({\text{T}}_{\text{CHEST}}+{\text{T}}_{\text{ARM}})+0.2\;({\text{T}}_{\text{THIGH}}+{\text{T}}_{\text{CALF}})$$

Ambient temperature was maintained at 20 to 23° C. All temperature probes were attached to two-channel electronic thermometers (Mallinckrodt model 6150, United States). Temperatures were recorded at 15-minute intervals. Mean body temperature (MBT) was calculated as:^21^


$$\text{MBT}=\left(0.66\text{x}{\text{T}}_{\text{CORE}}\right)+\left(0.34x\text{MST}\right)$$

Changes in total body heat content (TBHC) were expressed in kilojoules (kJ) as:^21^


$$\Delta \text{TBHC}=\Delta \text{MBT}({}^{\circ}\text{C})\;x\text{body weight}(\text{kg})\;x\;3.48\;(\text{kJ}{\text{.kg}}^{-1}.{}^{\circ}{\text{C}}^{-1})$$

where ΔMBT is the change in mean body temperature and 3.48 kJ.kg^-l^.°C^-l^) is the specific heat from the human body.

At admission to the operating room, the patients were randomly allocated via sealed envelope assignment to one of three groups, according to the preoperative and intraoperative thermal management. Ten patients did not receive preoperative or intraoperative active skin-surface warming (Gcontrol = control group). Ten patients received preoperative and intraoperative active skin-surface warming (Gpre+intra = preoperative + intraoperative warming group). Ten patients received intraoperative active warming (Gintra = intraoperative warming group). Before the operation, all patients in Gpre+intra were covered up to the shoulders with a forced-air warming blanket (WarmTouch model 5200, Mallinckrodt Medical, USA) that was set at 42 to 46° C for 45 minutes, before induction of anesthesia in the operating room. A cotton sheet was interposed between the skin and the blanket, which in turn was covered by one cotton sheet to reduce heat loss from the blanket to the environment. The preoperative management for the 20 patients in the unwarmed groups (Gcontrol and Gintra) consisted of passive thermal insulation with two double cotton sheets covering the body for 45 minutes, before induction of anesthesia in the operating room.

At the end of the preoperative warming for Gpre+intra, the active warming system was removed. Spinal anesthesia was then induced, with the patients in a seated position. A 26-G subarachnoid needle was inserted in the L_3_ - L_4_ interspace and 3 ml of 0.5% hyperbaric bupivacaine was injected. Patients were positioned on the operating room table, which was tilted as necessary to produce a bilateral T6 block (pinprick), and their skin was prepared with iodine before being covered with surgical drapes. For the Gpre+intra and Gintra patients, a forced-air warming blanket (Mallinckrodt Medical, USA) that was set at 42 to 46° C covered the thorax, shoulders, arms, and hands during the surgery. This blanket itself was covered by one cotton sheet to reduce heat loss to the environment. The temperature of the warming cover was reduced (to the range 36° C to 41° C) if the patient became uncomfortably warm or began to sweat. For the 10 patients in Gcontrol, two double cotton sheets covered the thorax, shoulders, arms, and hands during the surgery. No perioperative medication for sedation was utilized for the patients in any group.

At the end of the surgery, the active system was removed from the Gpre+intra and Gintra patients. Only using passive thermal insulation, the patients in all three groups were then immediately transferred to the Post-Anesthesia Care Room (PACU), where they were covered up to the shoulders with a WarmTouch blanket (Mallinckrodt Medical) that was set at 42 to 46° C, until their core temperature reached 36° C.

In the PACU, shivering was evaluated as absent, mild (when only detected as EKG artifacts) or severe (when clinically obvious), by an independent observer who was blinded to the study treatment used.

The sample size for the groups was estimated by using an expected difference of approximately 0.7 in the core temperature with a standard deviation of approximately 0.8 between Gcontrol and the other groups. The sample size for the groups was thus determined to be 10 patients. The power of the test used was 80%. In relation to type I and type II statistical errors, in calculating the sample size for the groups, we tried to use ways of minimizing these errors. Demographic variables, operating room temperature, mean skin, body and core temperatures, interval between the end of the prewarming period and the beginning of the surgery, duration of surgery, total infused fluids and time required to regain a core temperature of 36° C in the PACU were compared between the three groups by analysis of variance (ANOVA). The sexes were compared using Fisher's exact test. Continuous variables were compared between the groups using ANOVA for repeated measurements. All the data are presented as means ± standard deviation. For all analyses, p < 0.05 was used to determine statistical significance.

## RESULTS

The groups did not differ with regard to demographic variables and sex distribution (p > 0.05) (Table 1). The pre-induction operating room temperature, final operating room temperature, duration of surgery and total volume of IV fluids were similar in the three groups (p > 0.05) (Table 2).

**Table 1.** Distribution of gender and anthropometric variables for the three groups of patients that received pre operative midazolar

Group	Control group (n = 10)	Pre + intraoperative warming group (n = 10)	Intraoperative warming group (n = 10)
Age* (years)	39 ± 13	35 ± 13	41 ± 12
Heigh†* (cm)	168 ± 8	172 ± 8	173 ± 8
Weigh†* (kg)	71 ± 10	74 ± 12	71 ± 10
Body mass index* (kg.m^-2^)	23.6 ± 3.4	24.6 ± 2.8	23.4 ± 3.2
Sex† (M/F)	6/4	8/2	8/2

* *Data are means ± standard deviation (p > 0.10, by analysis of variance);*

† *Data are frequency distributions (p > 0.10, by Fisher’s exact test).*

**Table 2.** Intraoperative data for the three groups of patients that received preoperative midazolam

Group	Control group (n = 10)	Pre + intraoperative warming group (n = 10)	Intraoperative warming group (n = 10)
Preinduction operating room temperature (° C)	22.1 ± 0.8	22.2 ± 1.1	22.8 ± 0.4
Final operating room temperature (° C)	22.6 ± 0.7	22.7 ± 1.4	23.2 ± 0.4
Duration of surgery (min)	91 ± 32	98 ± 47	95 ± 25
Total fluid volume (ml)	1600 ± 718	1650 ± 529	1520 ± 571

*Data are expressed as means ± standard deviation. There were no differences between the groups (p > 0.10, by analysis of variance).*

**Figure 1:**
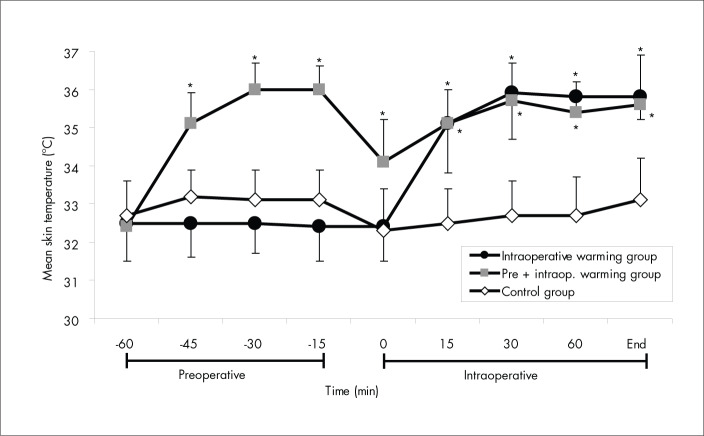
Mean skin temperature: changes in the preoperative and intraoperative periods for the three groups of patients that received preoperative midazolam. Data are presented as means ± standard deviation. *p < 0.05 *versus* control group (by analysis of variance).

**Figure 2:**
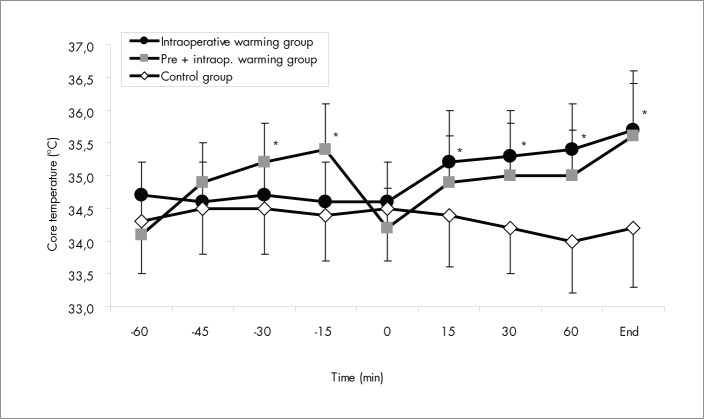
Core temperature: changes in the preoperative and intraoperative periods for the three groups of patients that received preoperative midazolam. Data are presented as means ± standard deviation. *p < 0.05 *versus* control group (by analysis of variance).

**Figure 3:**
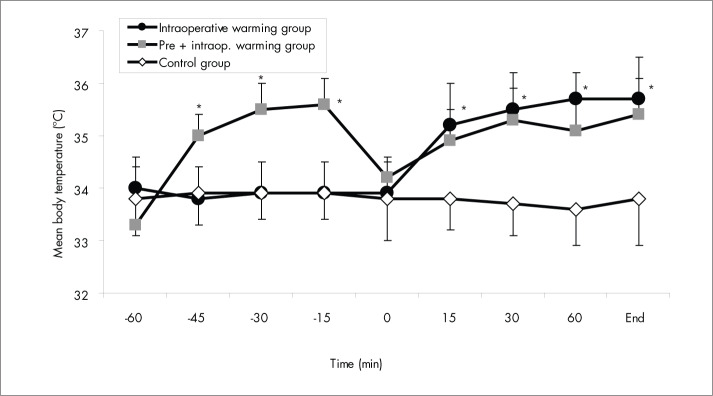
Mean body temperature: changes in the preoperative and intraoperative periods for the three groups of patients that received preoperative midazolam. Data are presented as means ± standard deviation. *p < 0.05 *versus* control group (by analysis of variance).

Mean skin, body, and core temperatures increased by 3.6° C, 2.3° C and 1.3° C, respectively, by the end of the 45 min of prewarming in Gpre+intra, whereas the body heat content gained during this phase was 543 ± 216 kJ. In Gcontrol and Gintra, the mean skin, body and core temperatures and the body heat content did not change significantly (p > 0.05). Mean skin, body and core temperatures (Figures 1, 2 and 3) and body heat content were significantly different between the unwarmed groups (Gcontrol and Gintra) and the warmed group (Gpre+intra) (p < 0.05) during this phase. All the patients in Gpre+intra assessed their prewarming as comfortable, with no sweating occurring during this phase.

Between the end of the prewarming period and the beginning of the surgery there was an interval of 9 ± 2 min, without any significant differences between the groups (p > 0.05). During this interval, for Gpre+intra, mean skin temperature decreased by 1.9° C, mean body temperature decreased by 1.4° C and core temperature decreased by 1.2° C. At the beginning of the surgery, among the groups, core and body temperatures were similar (p > 0.05) but not the mean skin temperature (p < 0.05). During the surgery, in Gpre+intra and Gintra, the mean skin, body and core temperatures increased significantly (p < 0.01), but only 50% of the patients in these groups presented normal temperatures (T_CORE_ > 36° C) at the end of surgery. In Gcontrol, the mean skin, body and core temperatures did not change significantly (p > 0.05) and all these patients were hypothermic at the end of the surgery. The mean skin, body and core temperatures were significantly different between the groups (p < 0.01) (Figures 1, 2 and 3) during the surgery.

The body heat content was significantly different between the groups (p < 0.05) during the surgery. In Gpre+intra and Gintra, body heat content increased significantly (p < 0.01) with forced-air warming during anesthesia, reaching 445 ± 223 kJ in Gintra and 329 ± 208 kJ in Gpre+intra at the end of the surgery. In Gcontrol, body heat content did not increase (p > 0.05) during anesthesia.

**Figure 4:**
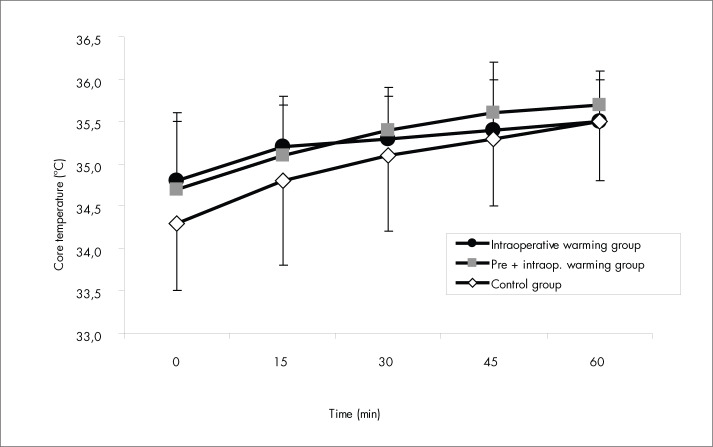
Core temperature: changes in the post-anesthesia care room for the three groups of patients that received preoperative midazolam. Data are presented as means ± standard deviation. p > 0.05 *versus* control group (by analysis of variance).

The mean spinal analgesia level was at the sixth thoracic level during surgery (T_6_ ± 1 segment) and decreased to T_10_ ± 1 segment at admission of the patients to the PACU. All patients in all three groups were in hypothermia (T_CORE_ < 36° C) at admission to the PACU. Between the end of the operation and the arrival of the patients in the PACU, the mean core temperature decreased by 0.9° C in both intraoperatively warmed groups (Gpre+intra and Gintra). In all groups, the core temperature increased significantly (p < 0.01) with forced-air warming, with no significant differences between the groups (p > 0.10) (Figure 4).

No patients in any of the three groups shivered in the operating room or PACU. No differences in the time required for regaining a core temperature of 36° C in the PACU were observed among the groups (p > 0.05): it was around 90 min for all three groups.

## DISCUSSION

In our study, the failure of preoperative warming plus intraoperative warming to maintain normal core temperature (36° C) in patients undergoing spinal anesthesia and surgery contrasted markedly with the effectiveness of this warming combination for preventing hypothermia during general anesthesia, as demonstrated in other studies.^14,15^ Possible impairment of thermoregulation, related to patients’ premedication with midazolam, might explain this phenomenon.^13,18,19^ The imbalance between heat production and cutaneous heat loss contributed little to core hypothermia after administration of midazolam.^22^ On the other hand, midazolam produced a concentration-dependent decrease in core temperature and impairment of tonic thermoregulatory vasoconstriction, thus allowing core-to-peripheral heat distribution in a dose-dependent manner at 30 minutes after IM injection.^18^

In addition, ambient temperature is a well-known predictor of core hypothermia in anesthetized patients. A study of typical ambient temperatures in operating rooms (20 to 23° C), showed that the mean frequency of core hypothermia (36° C) was approximately 50%.^2^ One limitation of our study was that, after midazolam premedication, all patients in all three groups lost their heat quickly and their core temperatures decreased before the start of the surgery. The basal core temperatures of all the patients in the three groups were around 34.5° C. This point may explain the lack of redistribution hypothermia in the unwarmed preoperative groups (Gcontrol and Gintra) because the patients were already hypothermic.

Redistribution of body heat is the major initial cause of hypothermia in patients who are administered spinal anesthesia.^23^ Redistribution during neuraxial anesthesia is typically restricted to the legs. The magnitude of this redistribution depends on the patient's initial thermal balance and can be minimized by active skin-surface warming before induction of neuraxial anesthesia.^16^ Preemptive skin-surface warming is effective and rapidly increases core temperature and body heat content, as demonstrated in our study and in other studies.^14–17^ Our gain in heat content through preinduction treatment (543 ± 216 kJ) is consistent with the findings from a previous study on heat storage in the human body.^17^

Spinal anesthesia inhibits thermoregulatory control centrally,^21^ but a far more important effect of neuraxial anesthesia is the blocking of peripheral sympathetic and motor nerves, which prevents thermoregulatory vasoconstriction and shivering.^5,24^ During spinal anesthesia, sufficient core hypothermia will trigger vasoconstriction and shivering in unblocked regions such as the arms,^19^ if thermoregulation is not excessively impaired by sedative medications.^1^ In our study, no patients shivered in the operating room or PACU. On the other hand, vasoconstriction that is limited to the upper body may be insufficient to prevent intraoperative hypothermia, but it does not seem to interfere with the efficiency of forced-air warming during spinal anesthesia and surgery, as showed in our study and in others.^l,25^

Rapid skin, body and core temperature decreases after inducing spinal anesthesia in Gpre+intra and during patient transfer to the PACU in the intraoperative warmed groups (Gpre+intra and Gintra) might be explained by the removal of the active warming system. This factor plays an important role, but it is not the only one. It seems that the combination of spinal anesthesia and midazolam administration obliterated any effective peripheral and central thermoregulatory control. On the other hand, these same factors had an important role in the rapid temperature increases that occurred as soon the warming system was activated during surgery and in the PACU. Residual spinal anesthesia, which maintained lower levels of body vasodilatation, seems to increase the rate of core rewarming faster than general anesthesia does.^1^ These points reinforce the usefulness of active skin-surface warming for patients during surgery and in the PACU. On the other hand, preoperative warming systems are less important when spinal anesthesia is used, because the gain in heat content through preinduction treatment is lost during the spinal anesthesia procedure, which is undertaken with the patients in a seated position. Our study also showed that passive thermal insulation of the patients did not minimize intraoperative hypothermia during spinal anesthesia.

## CONCLUSIONS

The addition of 45 minutes of preoperative skin-surface warming combined with intraoperative skin-surface warming does not avoid but minimizes hypothermia caused by spinal anesthesia, in patients with midazolam premedication. The combination of spinal anesthesia and midazolam administration seems to obliterate any effective peripheral and central thermoregulatory control, which causes rapid core temperature decrease after removal of the warming system.
